# Rational Design of Bifunctional Microporous Organic Polymers Containing Anthracene and Triphenylamine Units for Energy Storage and Biological Applications

**DOI:** 10.3390/ijms24108966

**Published:** 2023-05-18

**Authors:** Aya Osama Mousa, Zheng-Ian Lin, Cheng-Hsin Chuang, Chih-Kuang Chen, Shiao-Wei Kuo, Mohamed Gamal Mohamed

**Affiliations:** 1 Center of Crystal Research, Department of Materials and Optoelectronic Science, National Sun Yat-sen University, Kaohsiung 804, Taiwan; aya623514@gmail.com; 2 Institute of Medical Science and Technology, College of Medicine, National Sun Yat-sen University, Kaohsiung 804, Taiwan; chchuang@imst.nsysu.edu.tw; 3 Polymeric Biomaterials Laboratory, Department of Materials and Optoelectronic Science, National Sun Yat-sen University, Kaohsiung 804, Taiwan; chengyen0624@gmail.com (Z.-I.L.); chihkuan@mail.nsysu.edu.tw (C.-K.C.); 4 Department of Medicinal and Applied Chemistry, Kaohsiung Medical University, Kaohsiung 807, Taiwan; 5 Chemistry Department, Faculty of Science, Assiut University, Assiut 71515, Egypt

**Keywords:** microporous organic polymers, triphenylamine, pyrene, Suzuki cross-coupling reaction, electrochemical performance, MTT assay

## Abstract

In this study, we synthesized two conjugated microporous polymers (CMPs), An-Ph-TPA and An-Ph-Py CMPs, using the Suzuki cross-coupling reaction. These CMPs are organic polymers with p-conjugated skeletons and persistent micro-porosity and contain anthracene (An) moieties linked to triphenylamine (TPA) and pyrene (Py) units. We characterized the chemical structures, porosities, thermal stabilities, and morphologies of the newly synthesized An-CMPs using spectroscopic, microscopic, and N_2_ adsorption/desorption isotherm techniques. Our results from thermogravimetric analysis (TGA) showed that the An-Ph-TPA CMP displayed better thermal stability with T*_d10_* = 467 °C and char yield of 57 wt% compared to the An-Ph-Py CMP with T*_d10_* = 355 °C and char yield of 54 wt%. Furthermore, we evaluated the electrochemical performance of the An-linked CMPs and found that the An-Ph-TPA CMP had a higher capacitance of 116 F g^−1^ and better capacitance stability of 97% over 5000 cycles at 10 A g^−1^. In addition, we assessed the biocompatibility and cytotoxicity of An-linked CMPs using the MTT assay and a live/dead cell viability assay and observed that they were non-toxic and biocompatible with high cell viability values after 24 or 48 h of incubation. These findings suggest that the An-based CMPs synthesized in this study have potential applications in electrochemical testing and the biological field.

## 1. Introduction

The combustion of large quantities of petroleum fuels has caused environmental problems such as excessive CO_2_ pollution and hazardous particulate emissions from vehicle exhausts, leading to the pressing issue of climate change [1,2,3,4,5]. To address this issue, researchers have been exploring new and effective methods of renewable energy storage. One of the most practical approaches is the electrochemical energy storage system, which includes various devices based on electrochemistry concepts, such as batteries, conventional capacitors, and supercapacitors (SCs) [6,7,8,9,10,11,12,13,14]. Among these devices, SCs have gained significant attention due to their desirable features, including rapid charge/discharge kinetics, safety, high cycle stability, reasonable capacity intensity, low cost, environmentally friendly nature, outstanding lifespan, high power density, and high-rate efficiency [6,7,8,9,10,11,12,13,14]. Supercapacitors (SCs) have the potential to be used in a wide range of devices, including biomedical defibrillators, wind turbines, and hybrid buses and trams. SCs offer two different mechanisms for energy storage: non-faradaic methods, which involve the accumulation of electrostatic ionic charges at the electrode/electrolyte interface; and faradaic processes, which occur at the solid surface through reversible redox reactions [15,16,17,18,19,20]. The effectiveness of SCs depends on various factors, including the electrode material, which has gained significant attention in recent years due to its crucial role in SC performance [21,22,23]. Therefore, the development and production of new materials for SC electrodes have become a key research focus. Various types of materials have been used as electrodes for supercapacitors, including inorganic metal oxides, covalent triazine frameworks (CTFs), conjugated polymers, hyper-crosslinked polymers, hydroxides, doped carbon, sulfides, and covalent organic frameworks (COFs) [24,25,26,27,28,29,30,31,32,33,34,35]. However, inorganic materials have drawbacks such as resource scarcity and environmental pollution, highlighting the need for more affordable and environmentally friendly electrode materials [36]. Organic electroactive materials have emerged as a promising alternative due to their sustainability, environmental friendliness, flexibility, and resource availability. Among these, conjugated polymers (CPs) and conjugated microporous materials (CMPs) have been extensively studied as electrode materials for supercapacitors. CMPs, a subclass of organic polymers, have demonstrated outstanding electrochemical performance due to their physicochemical stability, facile preparation, pore structure variation, delocalized conjugation, and structural tunability [9,37,38,39,40,41]. CMP materials can be synthesized using a variety of building blocks and reactions [42,43], including oxidative polymerization, Schiff-base condensation reactions, and well-known coupling reactions such as Sonogashira-Hagihara, Yamamoto, and Suzuki-Miyaura [44,45,46,47,48,49,50,51,52]. These coupling processes typically result in high levels of polymerization and crosslinking and allow for the insertion of various organic moieties, leading to a wide range of possible CMP structures and characteristics. Aside from their various applications in energy and environmental fields, such as photocatalysis, dye adsorption, organic photovoltaic, gas adsorption, and metal ion sensing, conjugated microporous materials (CMPs) have also been extensively researched in the biomedical field for applications such as drug delivery, biosensing, phototherapy, and bioimaging, thanks to their unique properties [53,54,55,56,57,58,59,60,61,62,63,64,65,66]. However, for CMPs to be used in biomedical applications, they must be biocompatible. Therefore, we conducted an analysis of the in vitro cytotoxicity of the as-synthesized CMPs on mouse L929 fibroblast cells using an MTT assay and a live/dead cell viability assay. This study presents the synthesizing and characterizing of two new conjugated microporous polymers (CMPs) using one-pot polycondensation reactions. The two CMPs, An-Ph-TPA CMP and An-Ph-Py CMP, were based on TPA/Py and An units and were evaluated using several techniques such as FTIR, solid-state ^13^C NMR, TGA, XPS, BET, TEM, and SEM analyses. The results indicate that the CMPs are highly thermally stable and show promise for use in electrochemical applications. Specifically, the An-Ph-TPA CMP demonstrated higher electrochemical performance with an ultra-capacitance retention of up to 97% over 5000 cycles. Biocompatibility analysis using MTT assay and live/dead cell viability assay also demonstrated the potential for the CMPs to be used in biomedical applications. Overall, the rational design of An-linked CMPs not only allows for their use in electrical energy storage applications but also in biological fields due to their excellent biocompatibility.

## 2. Results and Discussion

### 2.1. Synthesis and Molecular Characterization of An-Linked CMPs (An-Ph-TPA and An-Ph-Py CMPs)

**Figure 1:**
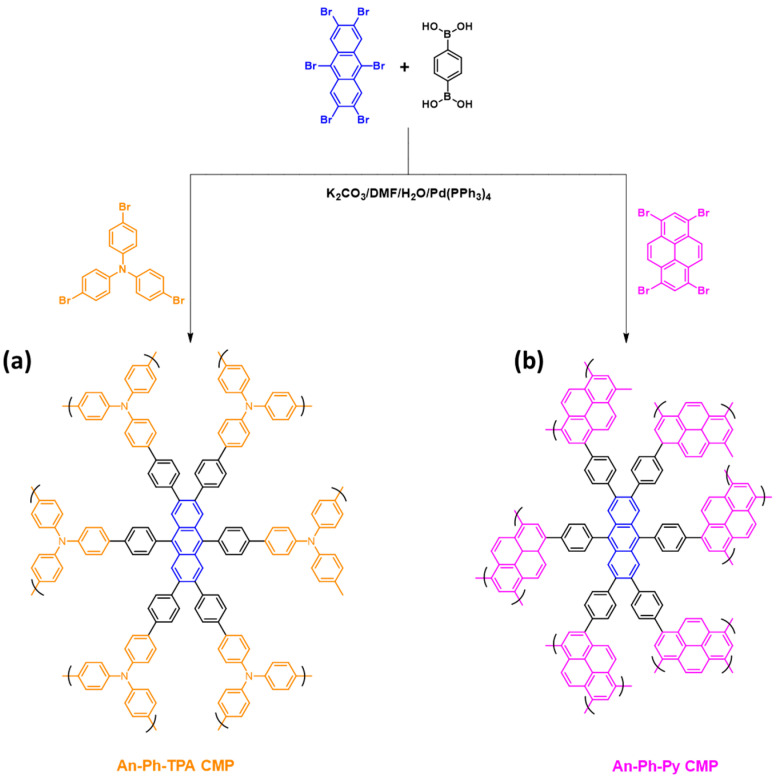
Schematic representation of the synthesis of (**a**) An-Ph-TPA CMP and (**b**) An-Ph-Py CMP.

**Figure 2:**
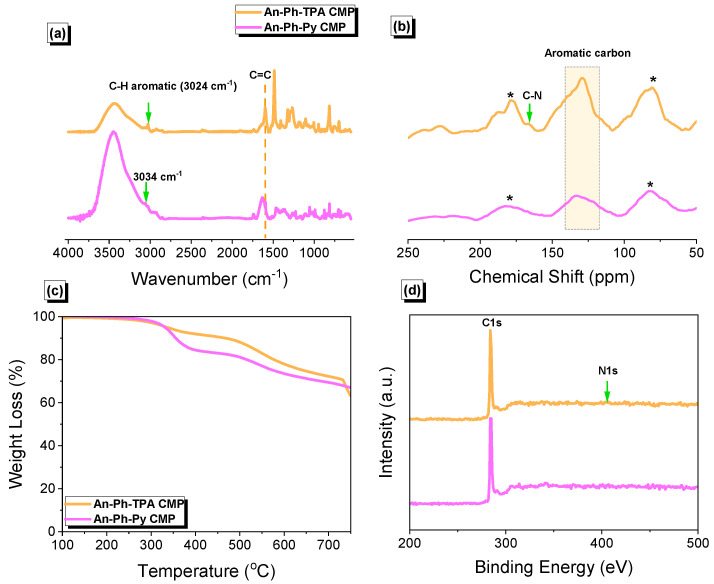
(**a**) FTIR spectra, (**b**) solid-state ^13^C NMR spectra, (**c**) TGA, and (**d**) XPS plots of An-Ph-TPA CMP and An-Ph-Py CMP. * is the side band of solid-state nuclear magnetic resonance spectroscopy (NMR).

Anthracene (An) and its derivatives are highly researched as conductive organic molecules due to their planar structure, which enables favorable long-range face-to-face arrangements through π-π interactions in resulting organic crystals [67,68,69,70]. This property makes them desirable for use in electronic applications. In addition to their conductive properties, anthracene and its derivatives have proven to be exceptional materials for organic electroluminescence [69,70]. Anthracene (An) was subjected to a bromination reaction in the presence of iron in CH_2_Cl_2_ to yield An-Br_6_ as a gray solid (Scheme S1). TPA-Br_3_ was synthesized as a white solid by reacting TPA with NBS in DMF (Scheme S2), while Py-Br_4_ was obtained as a green powder by refluxing Py with Br2 in the presence of nitrobenzene (Scheme S3). An-Br_6_, TPA-Br_3_, and Py-Br_4_ monomers were used as building blocks to synthesize two different CMPs. The reaction of An-Br_6_ with PhB(OH)_2_ and TPA-Br_3_ produced the An-Ph-TPA CMP, obtained as a green powder (Figure 1a). Similarly, the reaction of An-Br_6_ with PhB(OH)_2_ and Py-Br_4_ resulted in the An-Ph-Py CMP, which was also obtained as a green powder (Figure 1b). Both CMPs, which contained anthracene units, were found to be insoluble in all solvents, including THF, MeOH, H_2_O, CHCl_3_, DMF, EtOH, and acetone, indicating that they possessed highly cross-linked networks due to their high level of polymerization. FTIR measurements were conducted on the An-Br_6_, TPA-Br_3_, and Py-Br_4_ monomers. The FTIR spectra displayed peaks in the range of 3077–3054 and 1617–1527 cm^−1^, which corresponded to the stretching vibrations of aryl C-H bonds and C=C bonds. All spectroscopic data for An-Br_6_, TPA-Br_3_, and Py-Br_4_ monomers are presented in the Supporting Information (Figures S1–S6). The molecular structure, porosity properties, and thermal stability of An-Ph-TPA CMP and An-Ph-Py CMP were examined using various analytical techniques such as FTIR spectroscopy, solid-state ^13^C NMR, TGA, XPS, and BET. The FTIR spectra of An-Ph-TPA CMP and An-Ph-Py CMP (Figure 2a) showed absorption bands in the range of 3024–3034 and 1589 cm^−1^, corresponding to C-H aromatic and C=C bonds, which are characteristic of these materials. Solid-state ^13^C NMR analysis (Figure 2b) revealed carbon signals in the range of 147–114 ppm for both An-Ph-TPA CMP and An-Ph-Py CMP, assigned to the phenyl groups, along with a signal at 165.6 ppm corresponding to C-N bonds in An-Ph-Py CMP. TGA analyses (Figure 2c) showed that the An-Ph-TPA and An-Ph-Py CMPs exhibited weight losses of 10% under N_2_ atmospheres at temperatures of 467 and 355 °C, respectively. The char yields at 800 °C for An-Ph-TPA and An-Ph-Py CMPs were 57 and 54 wt%, respectively. XPS analysis (Figure 2d) confirmed the presence of C and N elements in An-Ph-TPA CMP and C elements in both An-Ph-TPA and An-Ph-Py CMPs. Overall, these results provide a comprehensive characterization of the molecular structure, porosity, and thermal stability properties of An-Ph-TPA CMP and An-Ph-Py CMP.

**Figure 3:**
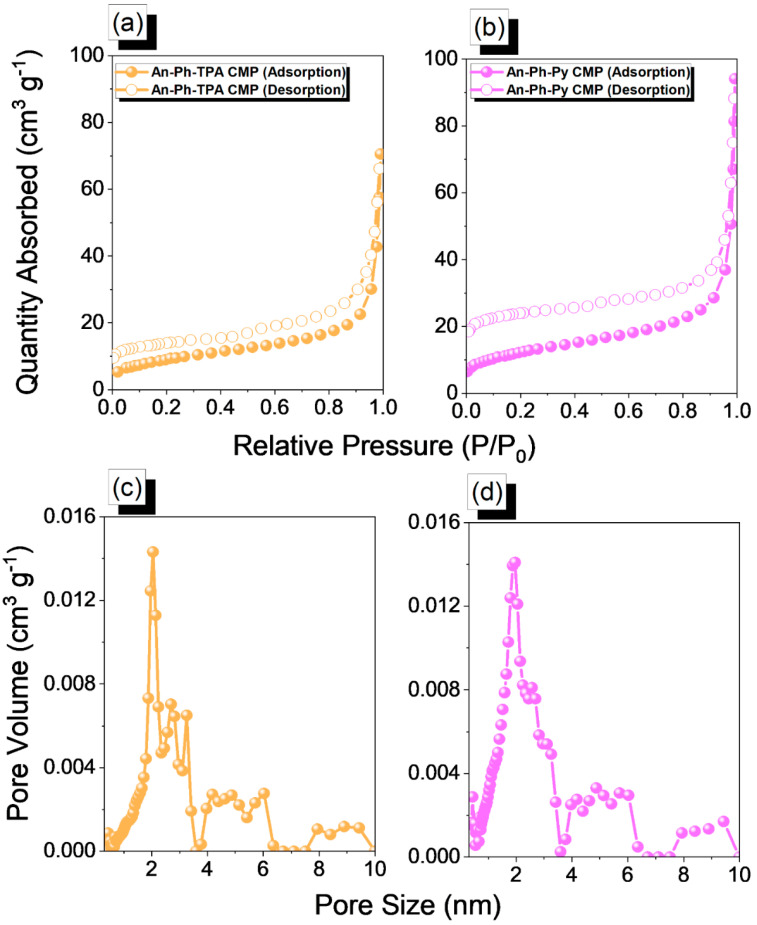
N_2_ adsorption/desorption isotherms and PSD curves of An-Ph-TPA CMP (**a**,**c**) and An-Ph-Py CMP (**b**,**d**).

To assess the porosity of An-Ph-TPA CMP and An-Ph-Py CMP, we investigated their N_2_ sorption behavior at 77 K. The adsorption-desorption profiles of both An-CMPs showed type I and IV characteristics (Figure 3a,b), with rapid N_2_ absorption in the low-pressure region, indicating that they are microporous materials. N_2_ adsorption increased at relative pressures between 0.1 and 0.8, and both isotherms showed constant nitrogen uptakes at P/P_0_ up to 0.8, suggesting the presence of mesopores and macropores in the two CMP structures. The BET surface areas and pore volume of An-Ph-TPA CMP were 33 m^2^ g^–1^ and 0.11 cm^3^ g^−1^, respectively, while those of An-Ph-Py CMP were 43.3 m^2^ g^−1^ and 0.15 cm^3^ g^−1^, respectively. The pore size distributions of An-Ph-TPA CMP and An-Ph-Py CMP (Figure 3c,d) were determined using nonlocal density functional theory (NLDFT) and showed main peaks at 1.94 and 1.92 nm, respectively, with additional peaks in the range of 2.65–9.34 nm for An-Ph-TPA CMP and 3.89–9.36 nm for An-Ph-Py CMP. Based on these results, both An-Ph-TPA CMP and An-Ph-Py CMP possess microporous and mesoporous architectures. Overall, these findings provide valuable insights into the porosity properties of An-Ph-TPA CMP and An-Ph-Py CMP.

**Figure 4:**
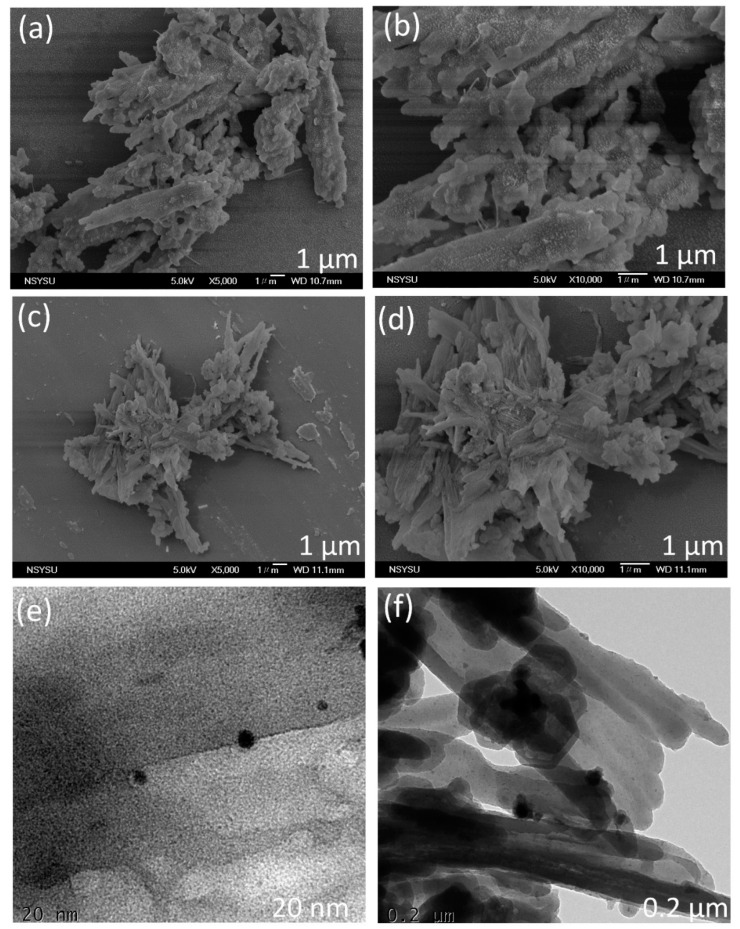
(**a**–**d**) SEM and (**e**,**f**) TEM images of the (**a**,**b**,**e**) An-Ph-TPA CMP and (**c**,**d**,**f**) An-Ph-Py CMP.

The morphology of the An-CMPs synthesized in this study was analyzed using high-resolution scanning electron microscopy (SEM) and transmission electron microscopy (TEM). The SEM images of An-Ph-TPA CMP showed an aggregated rod-like structure, while An-Ph-Py CMP displayed an aggregated tube-like structure (Figure 4a–d). The tubular morphology of An-Ph-Py CMP is attributed to the greater planarity of Py units, which promote their assembly in this form. TEM images of An-Ph-TPA CMP indicated the appearance of alternating light and dark regions, suggesting the existence of porous networks (Figure 4e). Similarly, the TEM image of An-Ph-Py CMP revealed a tube-like structure (Figure 4f).

**Figure 5:**
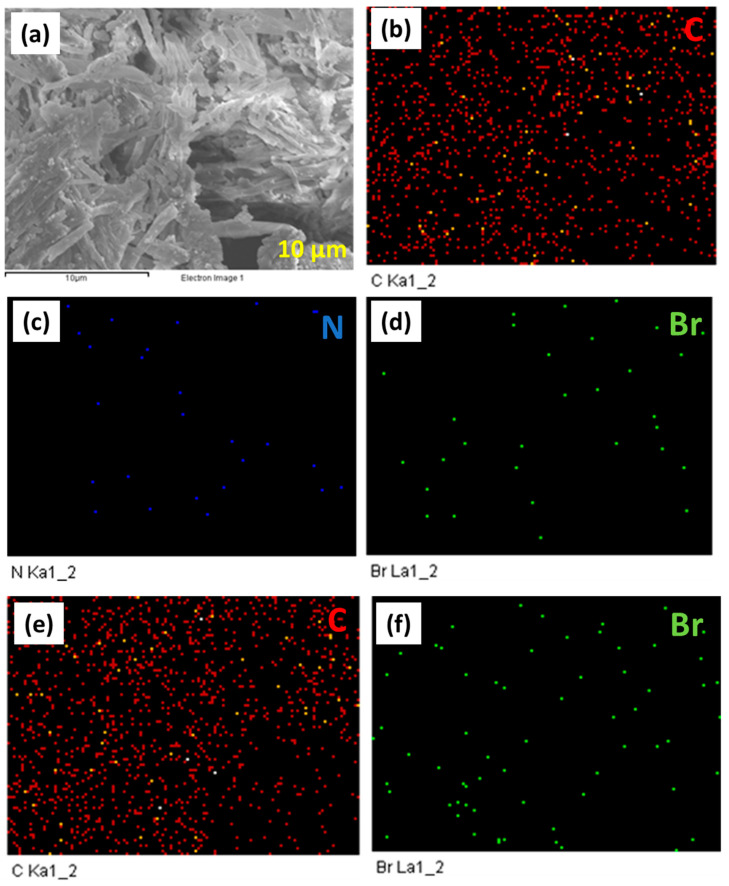
SEM-EDS image of An-Ph-TPA CMP (**a**) and EDS mapping of C (**b**), N (**c**), and Br (**d**) atoms in An-Ph-TPA CMP. EDS mapping of C (**e**) and Br (**f**) atoms in An-Ph-Py CMP. The scale bar in all images is 10 µm.

SEM-Elemental mapping (SEM-EDS) of An-Ph-TPA CMP (Figure 5a) provided evidence for the homogeneous distribution of carbon (Figure 5b), nitrogen (Figure 5c), and Br (Figure 5d) atoms in the An-Ph-TPA CMP skeleton. SEM-elemental mapping results exhibit the homogeneous distribution of carbon (Figure 5e) and Br (Figure 5f) atoms in the An-Ph-Py CMP framework. To gain a deeper understanding of the photophysical properties of the synthesized An-CMPs, we conducted photoluminescence (PL) and UV-visible measurements. The An-Ph-TPA CMP and An-Ph-Py CMP displayed UV-visible absorption peaks at 250 and 263 nm (Figure S7a), respectively, which can be attributed to specific electron transitions. The PL results indicated that the maximum PL fluorescence for An-Ph-TPA CMP and An-Ph-Py CMP was observed at 439 nm and 471 nm, respectively, when dispersed in absolute ethanol, as shown in Figure S7b. Additionally, An-Ph-TPA CMP and An-Ph-Py CMP displayed UV-visible absorption peaks at 250 and 263 nm, respectively, which can be attributed to specific electron transitions. These remarkable photophysical features suggest that An-CMPs can be used in photophysical-based applications, such as bioimaging.

### 2.2. Electrochemical Performance of An-Ph-TPA CMP and An-Ph-Py CMP

**Figure 6:**
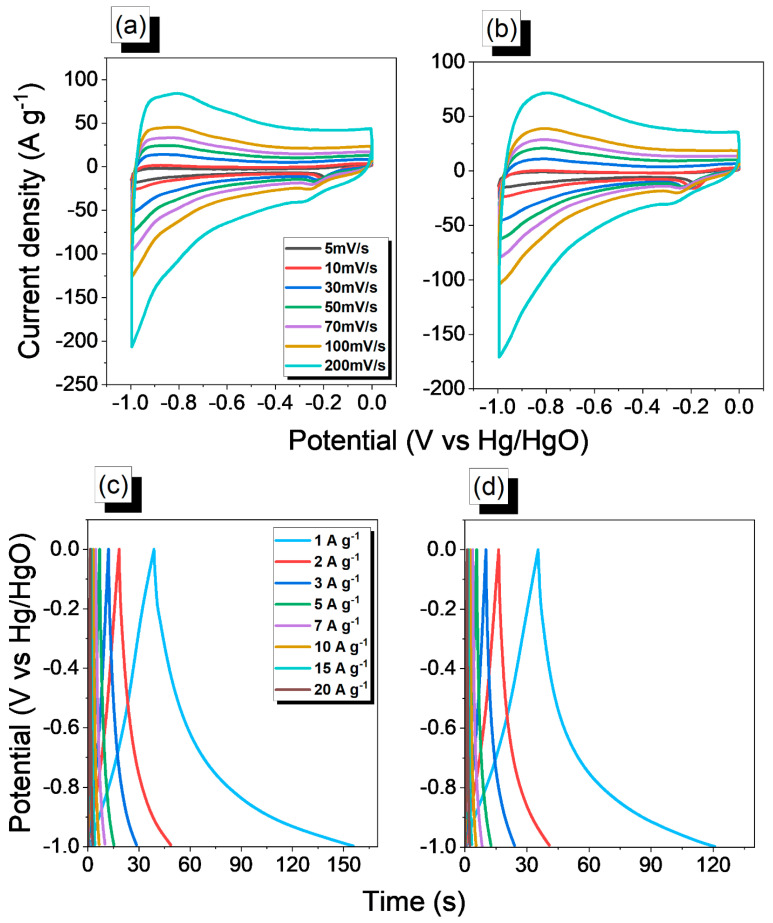
CV and GCD analyses of An-Ph-TPA CMP (**a**,**c**) and An-Ph-Py CMP (**b**,**d**).

The double-layer capacitive nature of the An-Ph-TPA and An-Ph-Py CMP samples was evaluated for their supercapacitive energy storage performance using a three-electrode supercapacitor system with 1 M KOH as the electrolyte. The cyclic voltammetry (CV) curves of the An-Ph-TPA and An-Ph-Py CMPs were recorded at different scan rates (5 to 200 mV s^−1^) between −1 and 0 V and exhibited a typical quasi-rectangular shape with good symmetry as depicted by Figure 6a,b. This finding suggests that the samples possess good double-layer capacitive characteristics. An investigation was conducted to assess the electrochemical capabilities of An-Ph-TPA and An-Ph-Py CMPs through GCD measurements. The results, displayed in Figure 6c,d, demonstrate that the GCD curves of the electrodes for both An-Ph-TPA and An-Ph-Py CMP display symmetrical triangular shapes with almost linear slopes, indicative of ideal electrochemical double-layer capacitors. These findings are consistent with the CV curves and provide further evidence of the double-layer capacitive nature of the An-Ph-TPA and An-Ph-Py CMPs framework.

**Figure 7:**
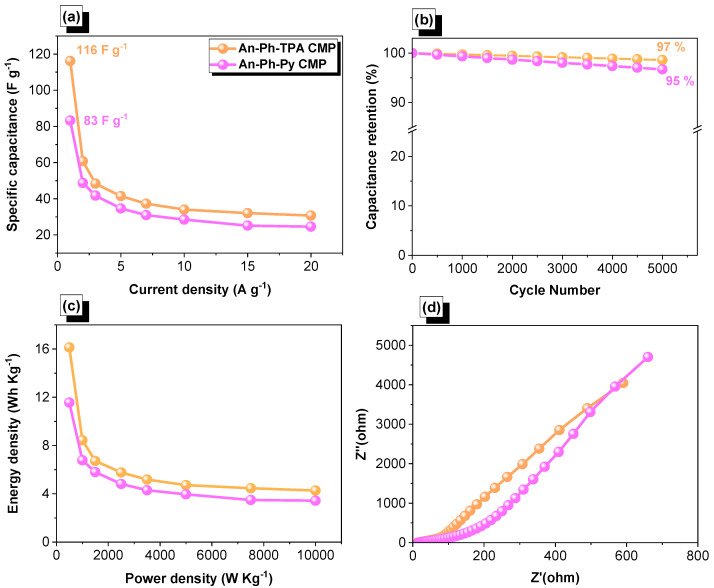
Capacitance (**a**), cycling stability (**b**), Ragone (**c**), and EIS (**d**) profiles of An-Ph-TPA CMP and An-Ph-Py CMP.

The specific capacitance, energy density, and power density of An-Ph-TPA CMP and An-Ph-Py CMP were calculated using the Equations (S1)–(S3). The An-Ph-TPA CMP showed specific capacitance values of 116, 61, 49, 42, 37, 34, 32, and 31 F g^−1^ at current densities of 1, 2, 3, 5, 7, 10, 15, and 20 A g^−1^, respectively. In comparison, the An-Ph-Py CMP showed specific capacitance values of 83, 49, 42, 35, 31, 29, 26, and 25 F g^−1^ at the same corresponding current densities (Figure 7a). Therefore, the An-Ph-TPA CMP demonstrated higher capacitance values than the An-Ph-Py CMP at all measured current densities. The improved electrochemical performance of the An-Ph-TPA CMP framework can be attributed to the existence of a more extensive conjugated system and the incorporation of TPA units. The capacitive retention of both An-Ph-TPA and An-Ph-Py CMP frameworks was determined through GCD measurements, with values of 97% and 95%, respectively Figure 7b. These values indicate that the An-Ph-TPA CMP framework has slightly higher capacitive retention than the An-Ph-Py CMP framework. Furthermore, specific capacitances of An-Ph-TPA CMP (116 F g^−1^) and An-Ph-Py CMP (83 F g^−1^) are higher than TPA-Bz CMP (55.1 F g^−1^) [71], Pyra-BP-HPP (94 F g^−1^) [71], Py-BSU CMP (38 F g^−1^) [72], TBN-BSU CMP (70 F g^−1^) [72], TBN-TPE-CMP (18.45 F g^−1^) [73], TBN-Car-CMP (18.90 F g^−1^) [73], TBN-Py-CMP (31 F g^−1^) [73], and CoPc-CMP(13.7 F g^−1^) [36]. The Ragone plot comparison between An-Ph-TPA and An-Ph-Py CMP frameworks reveals that the former exhibits a maximum energy density of 16 W h kg^−1^, which is twice as high as that of the latter, which is only 12 W h kg^−1^ when the power density is 500 kW kg^−1^, as shown in Figure 7c. The notable enhancement in specific capacitance and energy density observed in An-Ph-TPA CMP can be attributed to the superior electron transportation ability and higher ion diffusion rate facilitated by the TPA moiety present in the An-Ph-TPA CMP sample. Electrochemical Impedance Spectroscopy (EIS) is a technique that is widely used to investigate the electrochemical characteristics of materials, including supercapacitors. EIS data provide information about the system’s impedance at different frequencies, as well as other electrochemical parameters like capacitance and resistance. The EIS data show that the ohmic resistance of An-Ph-Py CMP and An-Ph-TPA-CMP were 3.259 ohms and 1.729 ohms, respectively, as depicted in Figure 7d. A higher ohmic resistance indicates that the flow of current through the system is more difficult, which can result in lower supercapacitor performance. Therefore, An-Ph-TPA CMP exhibited superior electrochemical performance compared to its counterpart, An-Ph-Py CMP.

### 2.3. Cytotoxicity Assessment of An-Ph-TPA CMP and An-Ph-Py CMP

**Figure 8:**
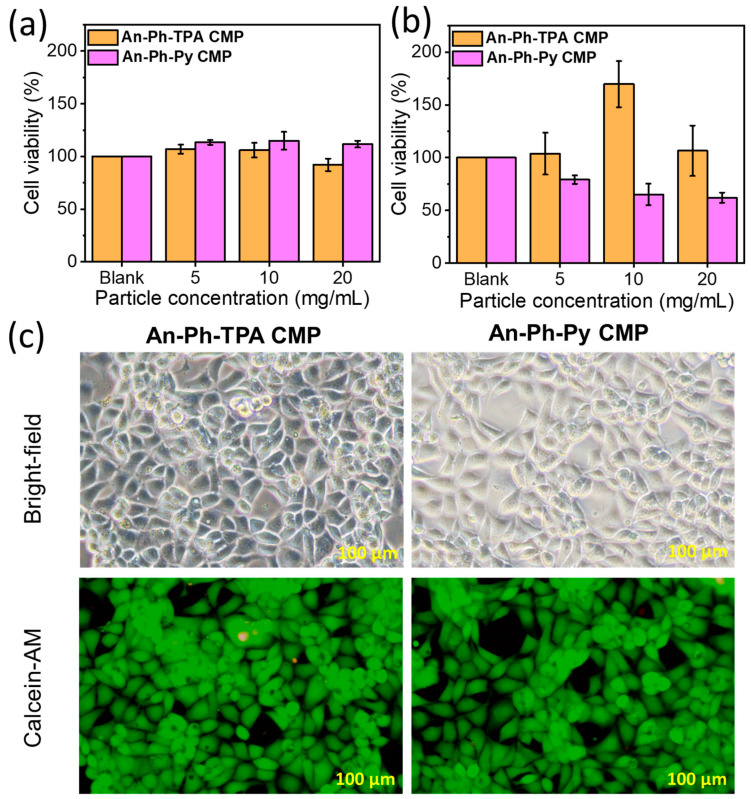
Cytotoxicity assessment of An-Ph-TPA CMP and An-Ph-Py CMP on L929 fibroblasts after (**a**) 24 h and (**b**) 48 h of culture; (**c**) fluorescence images of L929 fibroblasts (Calcein-AM) after 48 h of culture.

In order to determine the potential cytotoxicity of An-Ph-TPA CMP and An-Ph-Py CMP, L929 mouse fibroblasts were used as test cells. However, since An-linked CMPs are insoluble, their cytotoxicity was assessed using the ISO 10993-5 standard screening method. The An-CMPs were immersed in a cell culture medium for 24 h. The viability of L929 cells was then evaluated using an MTT test after 24 and 48 h of incubation with the CMP. The results showed that cell survival parameters were over 90% for all concentrations of extract medium, indicating that An-linked CMPs had extremely low hazard toxicity to L929 cells, as depicted in Figure 8. To further confirm these findings, a live/dead cell viability assay was performed, which showed a significant increase in the growth of L929 mouse cells. Therefore, An-linked CMPs are highly biocompatible and nontoxic, making them safe for use in biomedical and biological applications. Figure 8a,b shows the cell compatibility of An-Ph-TPA CMP and An-Ph-Py CMP with L929 cells at different concentrations after 24 and 48 h of culture. According to the experimental results, the cell survival rate of An-Ph-TPA CMP and An-Ph-Py CMP at concentrations of 5, 10, and 20 mg/mL is over 80%. Furthermore, after 48 h of culture, An-Ph-TPA CMP can promote L929 cell proliferation (cell compatibility over 100%), indicating that this material can promote fibroblast proliferation. However, the cell compatibility of An-Ph-Py CMP is over 70%, and, according to ISO 10993-5, both An-Ph-TPA CMP and An-Ph-Py CMP show no cell toxicity. These MTT results suggest that An-Ph-TPA CMP and An-Ph-Py CMP are non-toxic to L929 fibroblasts and can be considered highly biologically safe and compatible biomaterials. In addition to confirming negligible cell toxicity, this study used Calcein-AM to stain L929 cells and observe cell survival using fluorescence microscopy. Figure 8c shows optical microscopy (OM) and fluorescence microscopy images of An-Ph-TPA CMP and An-Ph-Py CMP co-cultured with L929 cells for 48 h. The OM results show that L929 cells display a good spreading morphology and a large number of cells. Furthermore, the fluorescence microscopy image shows that green fluorescence represents live cells, and based on the fluorescence intensity, it can be inferred that a large number of L929 cells have successfully proliferated.

## 3. Experimental Part

### 3.1. Materials

Anthracene (An), bromine (Br_2_), iron powder, dichloromethane (CH_2_Cl_2_), tetrakis(triphenylphosphine) palladium (Pd(PPh_3_)_4_), nitrobenzene (C_6_H_5_NO_2_), Pyrene (Py), brine, ethanol (EtOH), benzene-boronic acid [PhB(OH)_2_], anhydrous magnesium sulfate (MgSO_4_, 99.5%), tetrahydrofuran (THF), sodium thiosulfate (Na_2_S_2_O_3_), N-bromosuccinimide (NBS, 99%), dimethylformamide (DMF), triphenylamine (TPA), methanol (MeOH), and potassium carbonate (K_2_CO_3_, 99.9%) were ordered from Sigma Aldrich and Alfa Aesar.

### 3.2. Synthesis of 2,3,6,7,9,10-Hexabromoanthracene (An-Br_6_)

To prepare An-Br6, a mixture of 795 mg (4.5 mmol) of anthracene, 1.64 g (30 mmol) of bromine, and 1.68 g (30 mmol) of iron powder was combined with 30 mL of dry CH_2_Cl_2_ in a round-bottom flask and stirred under argon at room temperature for 6 h. The resulting mixture was then quenched with 10% Na_2_S_2_O_3_ and extracted twice with 75 mL of CH_2_Cl_2_. The obtained organic extracts were then washed with brine, water, and MeOH, and the obtained powder was dried in an oven. This process yielded a gray solid weighing 2.04 g (71% yield based on Scheme S1). FTIR (Figure S1): 3077, 1527, and 588 cm^−1^. The ^1^H and ^13^C NMR data of An-Br_6_ cannot provide because of its poor solubility in all organic solvents.

### 3.3. Synthesis of Tris(4-bromophenyl)amine (TPA-Br_3_)

In a round-bottom flask, 3 g (17.25 mmol) of NBS was added to a solution of 1.37 g (5.69 mmol) of TPA in 45 mL of DMF. The resulting mixture was stirred for 24 h at 0 °C, then extracted with CH_2_Cl_2_ and H_2_O, and the DMF was evaporated. The organic layer obtained was dried over MgSO4, filtered, and washed multiple times with MeOH to obtain TPA-Br_3_ as a white powder, with a yield of 2.81 g (90%) based on Scheme S2. M.p.: 142 °C (Figure S2). FTIR (Figure S3): 3078, 1618 (C=C stretching). ^1^H NMR (Figure S4): 6.94–7.35 (12H). ^13^C NMR (Figure S5): 146.80–116.40.

### 3.4. Synthesis of 1,3,6,8-Tetrabromopyrene (Py-Br_4_)

To prepare Py-Br_4_, a solution of Br_2_ (3.45 mL, 66 mmol) was added to 30 mL of C_6_H_5_NO_2,_ and the resulting solution was added to a flask containing a solution of pyrene (3.00 g, 15 mmol) in 30 mL of nitrobenzene. The mixture was then refluxed for four hours at 120 °C until a green solid was formed. The resulting solid was washed with ethanol, filtered, and dried, yielding Py-Br_4_ (Scheme S3) with a yield of 90% (6.6 g). The FTIR spectrum (Figure S6) of Py-Br_4_ showed peaks at 3054 cm^−1^, corresponding to aromatic C-H stretching, and 681 cm^−1^, corresponding to C-Br stretching. The ^1^H and ^13^C NMR of Py-Br_4_ don’t provide because of its poor solubility in all organic solvents.

### 3.5. Preparation of An-Ph-TPA and An-Ph-Py CMPs

To synthesize An-Ph-TPA and An-Ph-Py CMPs, a solution of An-Br_6_ (0.3 g, 0.46 mmol), PhB(OH)_2_ (0.23 g, 1.38 mmol), either TPA-Br_3_ (0.119 g, 0.25 mmol) or Py-Br_4_ (0.119 g, 0.23 mmol), k_2_CO_3_ (0.51 g, 3.69 mmol), and Pd(PPh_3_)_4_ (0.05 g, 0.04 mmol) in 20 mL of DMF was refluxed at 90 °C for three days. The resulting green powder of An-Ph-TPA and green solid of An-Ph-Py CMPs were filtered and repeatedly washed with THF, water, methanol, and acetone. The obtained green powders of An-Ph-TPA and An-Ph-Py CMPs were then dried overnight at 100 °C. The syntheses of these compounds are displayed in Figure 1, respectively.

### 3.6. Cell Viability via an MTT Assay

Determining the cytotoxicity of compounds is crucial in investigating their potential antimicrobial applications. In this study, L929 fibroblast cells obtained from ATTCC (CCL-1) were selected as the research cells to evaluate the cytotoxicity of An-CMPs samples. The L929 cell line was cultured in DMEM/F-12 supplemented with 10% fetal bovine serum and 1% penicillin-streptomycin. To extract the An-CMPs, they were incubated in DMEM/F-12 at 37 °C for 24 h, and the resulting extract medium was utilized as a cultured medium for L929 cells. The cells were seeded in a 96-well plate with a cell population of 6 x 10^6^ cells per well and cultured at 37 °C with 5% CO_2_ for 24 h. After seeding and cultivation, the extract medium was added to each well and cultured for 24 and 48 h. Subsequently, the extract medium was removed, and the cells were washed with PBS solution at a pH of 7.4. Then, 10 µL of MTT solution (1 mg/1 mL in DMEM/F-12) was added to each well, and the plate was incubated for 4 h at 37°C with 5% CO_2_. The medium in each well was then removed, and 100 µL of dimethyl sulfoxide (DMSO) was added to decompose the formed formazan reaction products. The plate was shaken gently for 15 min to ensure complete dissolution in DMSO. The optical density of the solution in each well was measured at 450 nm using a microplate reader (800TS/BioTek), with 630 nm serving as the reference wavelength.

## 4. Conclusions

In this study, we successfully synthesized two types of CMPs, An-Ph-TPA CMP and An-Ph-Py CMP, containing An moieties through the Suzuki cross-coupling reaction, which were fully characterized by various analytical techniques such as FTIR, BET, solid-state ^13^C NMR spectroscopy, TEM, and SEM. Both CMPs demonstrated high thermal stability with T*_d10_* up to 467 °C and char yield up to 57 wt% at 800 °C as evaluated by TGA. Importantly, electrochemical measurements revealed that the An-Ph-TPA CMP exhibited a higher specific capacitance of up to 116 F g^−1^, with ultra-capacitance retention of up to 97% over 5000 cycles, compared to another porous polymer. The incorporation of triphenylamine (TPA) units into the An-Ph-TPA CMP, compared to the pyridine-based moiety of An-Ph-Py CMP, was suggested to be responsible for the higher capacitance of the former. In addition, we assessed the biocompatibility and cytotoxicity of the An-linked CMPs towards mouse L929 fibroblasts cells using MTT assay and live/dead cell viability assay, which revealed that the viability of L929 cells was higher than 90%, indicating that the An-linked CMPs were non-toxic and extremely biocompatible. This study not only provides new CMPs with high capacitances but also highlights the potential of using anthanthrene-based materials in energy storage and biological applications through molecular engineering.

## Data Availability

The data presented in this study are available on request from the corresponding author.

## Supplementary Materials

The supporting information can be downloaded at: https://www.mdpi.com/article/10.3390/ijms24108966/s1.
