# Supplementary material for: Rational Design of Bifunctional Microporous Organic Polymers Containing Anthracene and Triphenylamine Units for Energy Storage and Biological Applications
Source: Int J Mol Sci. 2023 May 18;24(10):8966. doi: 10.3390/ijms24108966 (PMC10218919; doi:10.3390/ijms24108966)
Supplement: Supplementary file 1 [file ijms-24-08966-s001.zip › ijms-2369098-supplementary.pdf]

Rational Design of Bifunctional Microporous Organic Polymers Containing Anthracene and Triphenylamine Units for Energy Storage and Biological Applications

Aya Osama Mousa [1,2], Zheng-Ian Lin [3], Cheng-Hsin Chuang [2], Chih-Kuang Chen [3], Shiao-Wei Kuo [1,4,*] and Mohamed Gamal Mohamed [1,5,*]

[1] Center of Crystal Research, Department of Materials and Optoelectronic Science, National Sun Yat-sen University, Kaohsiung 804, Taiwan; aya623514@gmail.com
[2] Institute of Medical Science and Technology, College of Medicine, National Sun Yat-sen University, Kaohsiung 804, Taiwan; chchuang@imst.nsysu.edu.tw
[3] Polymeric Biomaterials Laboratory, Department of Materials and Optoelectronic Science, National Sun Yat-sen University, Kaohsiung 804, Taiwan; chengyen0624@gmail.com (Z.-I.L.); chihkuan@mail.nsysu.edu.tw (C.-K.C.)
[4] Department of Medicinal and Applied Chemistry, Kaohsiung Medical University, Kaohsiung 807, Taiwan
[5] Chemistry Department, Faculty of Science, Assiut University, Assiut 71515, Egypt
* Correspondence: kuosw@faculty.nsysu.edu.tw (S.-W.K.); mgamal.eldin34@gmail.com (M.G.M.)

Characterization

FTIR spectra were collected on a Bruker Tensor 27 FTIR spectrophotometer with a resolution of 4 cm^{-1} by using KBr disk method. ^{13}C nuclear magnetic resonance (NMR) spectra were examined by using an INOVA 500 instrument with DMSO as the solvent and TMS as the external standard. Chemical shifts are reported in parts per million (ppm). The thermal stabilities of the samples were performed by using a TG Q-50 thermogravimetric analyzer under a N_2 atmosphere; the cured sample (ca. 5 mg) was put in a Pt cell with a heating rate of 20 °C min^{-1} from 100 to 800 °C under a N_2 flow rate of 60 mL min^{-1}. Wide-angle X-ray diffraction (WAXD) patterns were measured by the wiggler beamline BL17A1 of the National Synchrotron Radiation Research Center (NSRRC), Taiwan. A triangular bent Si (111) single crystal was used to get a monochromated beam having a wavelength (λ) of 1.33 Å. The morphologies of the polymer samples were examined by Field emission scanning electron microscopy (FE-SEM; JEOL JSM7610F) and also by transmission electron microscope (TEM) using a JEOL-2100 instrument at an accelerating voltage of 200 kV. BET surface area and porosimetry measurements of

samples (ca. 40–100 mg) were measured using BEL Master^{TM}/BEL sim^{TM} (v. 3.0.0). N_2 adsorption and desorption isotherms were generated through incremental exposure to ultrahigh-purity N_2 (up to ca. 1 atm) in a liquid N_2 (77 K) bath. Surface parameters were calculated using BET adsorption models in the instrument’s software. The prepared samples' pore size was determined using nonlocal density functional theory (NLDFT).

Electrochemical Analysis

Working Electrode Cleaning: Prior to using, the glassy carbon electrode (GCE) was polished several times with 0.05-μm alumina powder, washed with EtOH after each polishing step, cleaned through sonication (5 min) in a water bath, washed with EtOH, and then dried in the oven at 50 °C.

Electrochemical Characterization: The electrochemical experiments were performed in a three-electrode cell using an Autolab potentiostat (PGSTAT204) and 1 M KOH as the aqueous electrolyte. The GCE was used as the working electrode (diameter: 5.61 mm; 0.2475 cm^2); a Pt wire was used as the counter electrode; Hg/HgO (RE-1B, BAS) was the reference electrode. All reported potentials refer to the Hg/HgO potential. A slurry was prepared by dispersing An-Ph-TPA CMP or An-Ph-Py CMP (45 wt %), carbon black (45 wt %), and Nafion (10 wt%) in a mixture of (EtOH/ H_2O) (200 μL: 800 μL) and then sonicating for 1 h. A portion of this slurry (10 μL) was pipetted onto the tip of the electrode, which was then dried in air for 30 min prior to use. The electrochemical performance was studied through CV at various sweep rates (5–200 mV s^{-1}) and through the GCD method in the potential range from 0 to -1.00 V (vs. Hg/HgO) at various current densities (0.5–20 A g^{-1}) in 1 M KOH as the aqueous electrolyte solution.

The specific capacitance was calculated from the GCD data using the equation.

$$C_s = (I\Delta t)/(m\Delta V) \qquad \textbf{(S1)}$$

Where C_s (F g^{-1}) is the specific capacitance of the supercapacitor, I (A) is the discharge current, ΔV (V) is the potential window, Δt (s) is the discharge time, and m (g) is the mass of the NPC on the electrode. The energy density (E, W h kg^{-1}) and power density (P, W kg^{-1}) were calculated using the equations.

$$E = 1000C(\Delta V)^2/(2 \times 3600) \quad \textbf{(S2)}$$

$$P = E/(t/3600) \quad \textbf{(S3)}$$

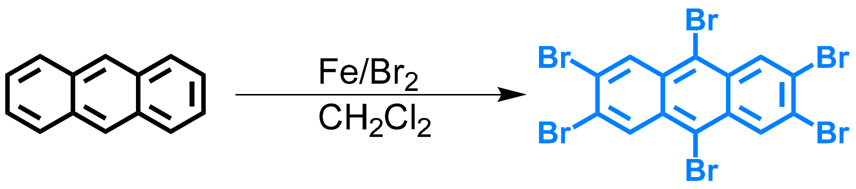

Scheme S1. Synthesis of An-Br_6.

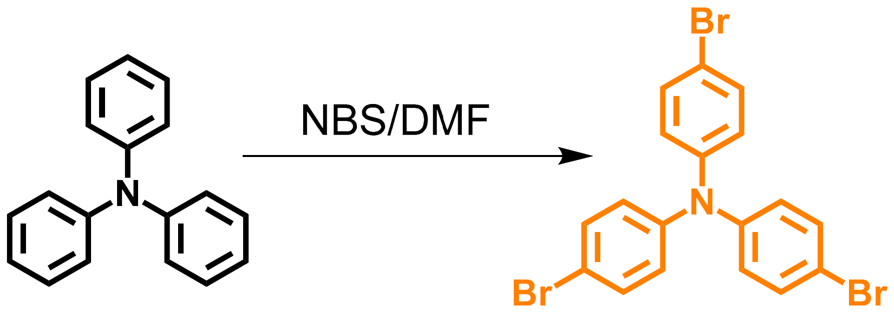

Scheme S2. Synthesis of TPA-Br_3.

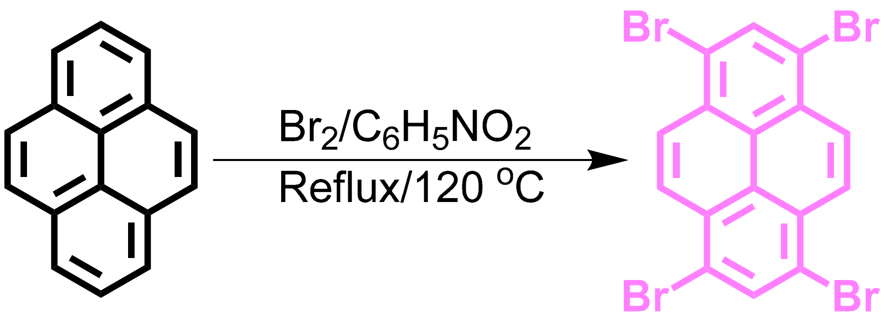

Scheme S3. Synthesis of Py-Br_4.

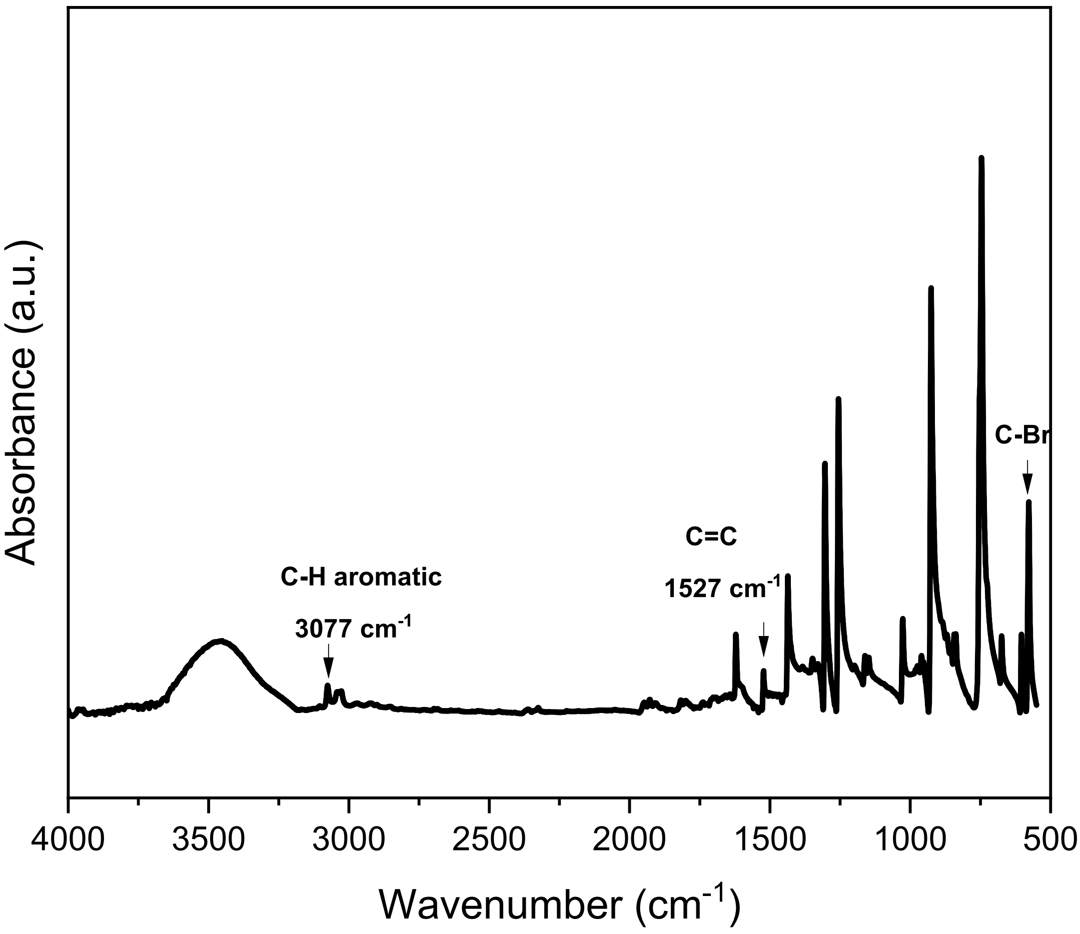

Figure S1. FT-IR spectrum of An-Br_6.

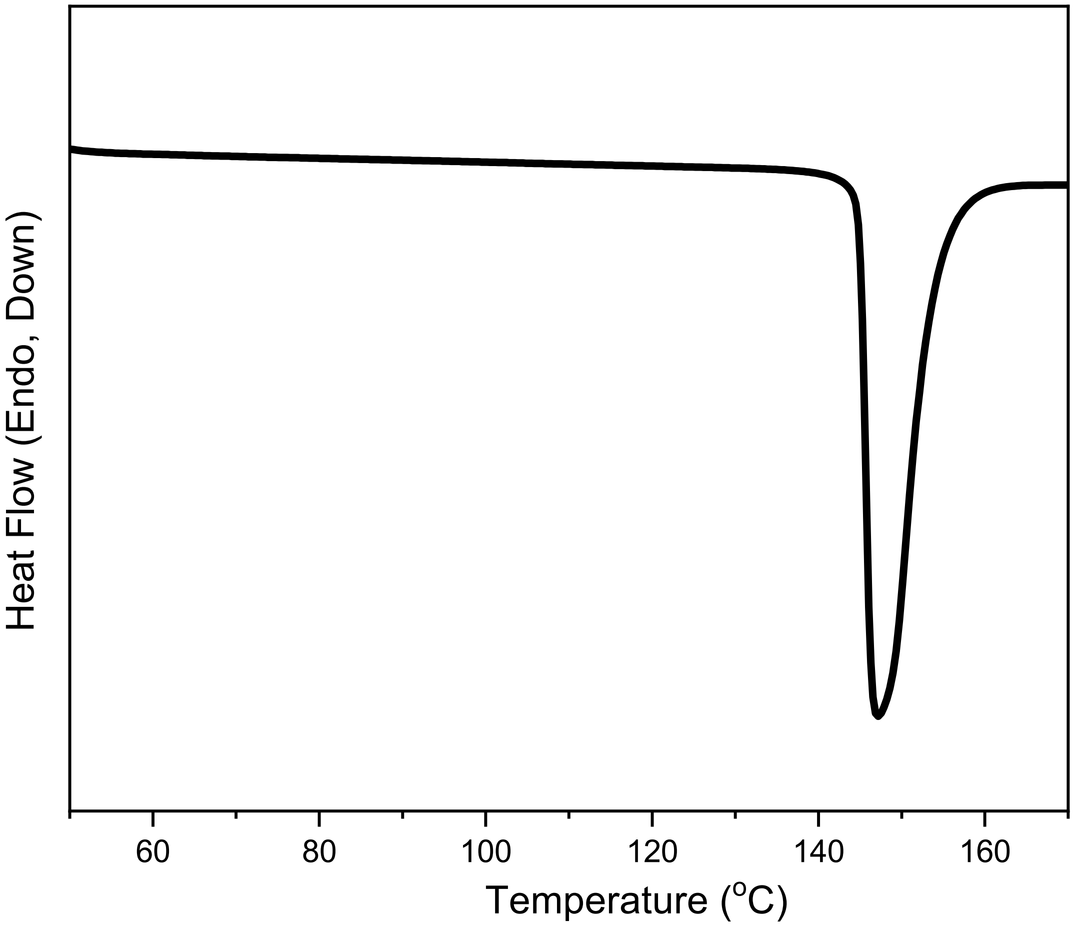

Figure S2. DSC profile of TPA-Br_3.

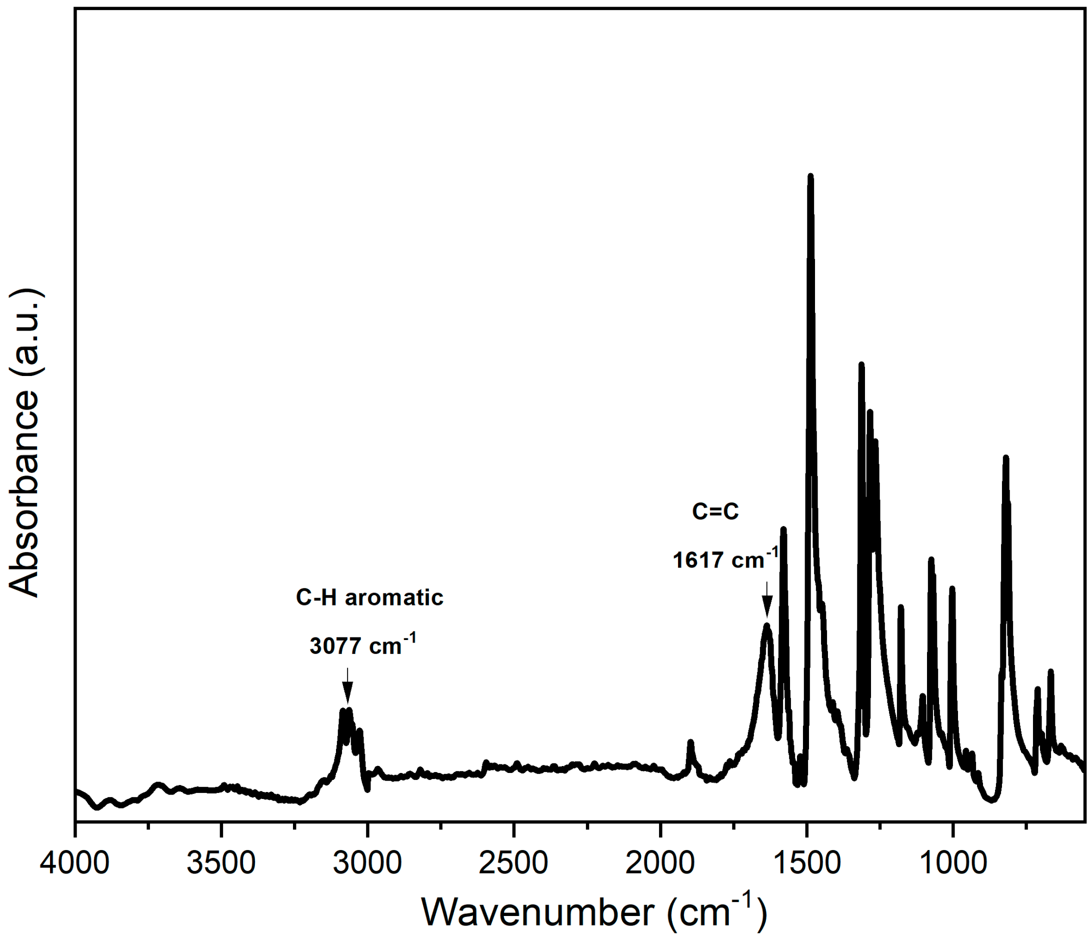

Figure S3. FTIR profile of TPA-Br_3.

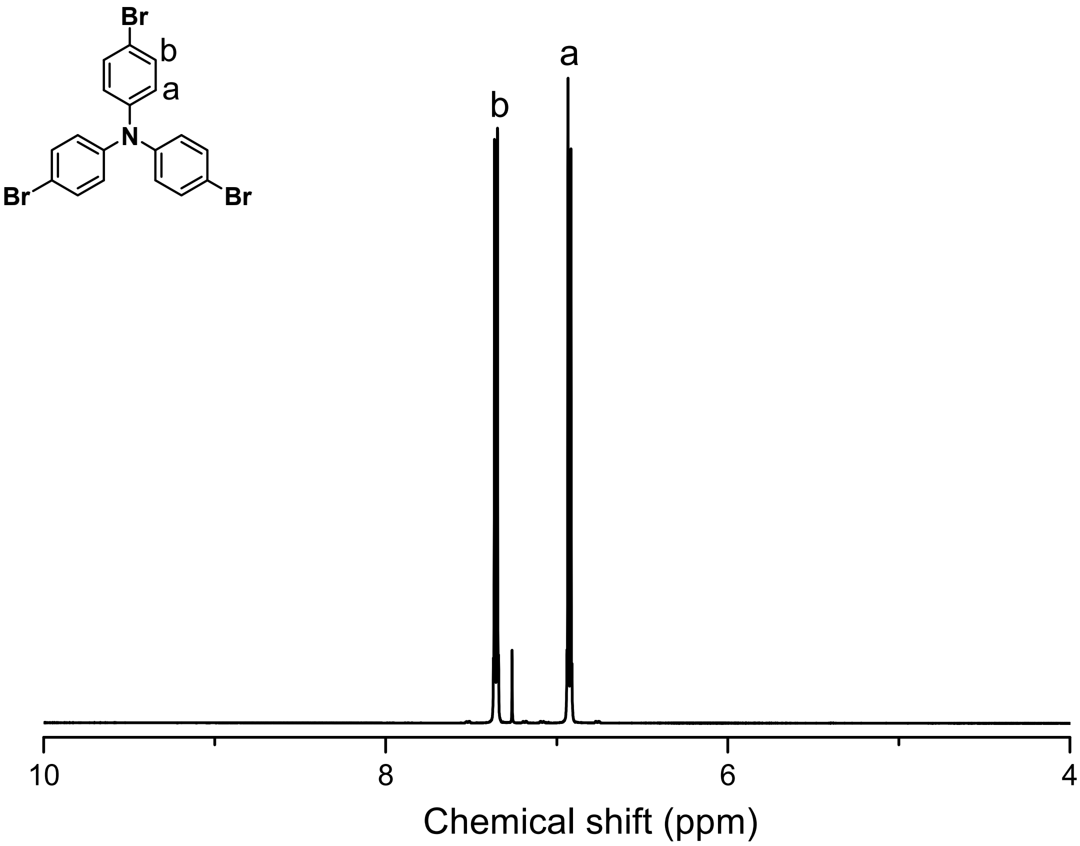

Figure S4. ^{1}H NMR spectrum of TPA-Br_3.

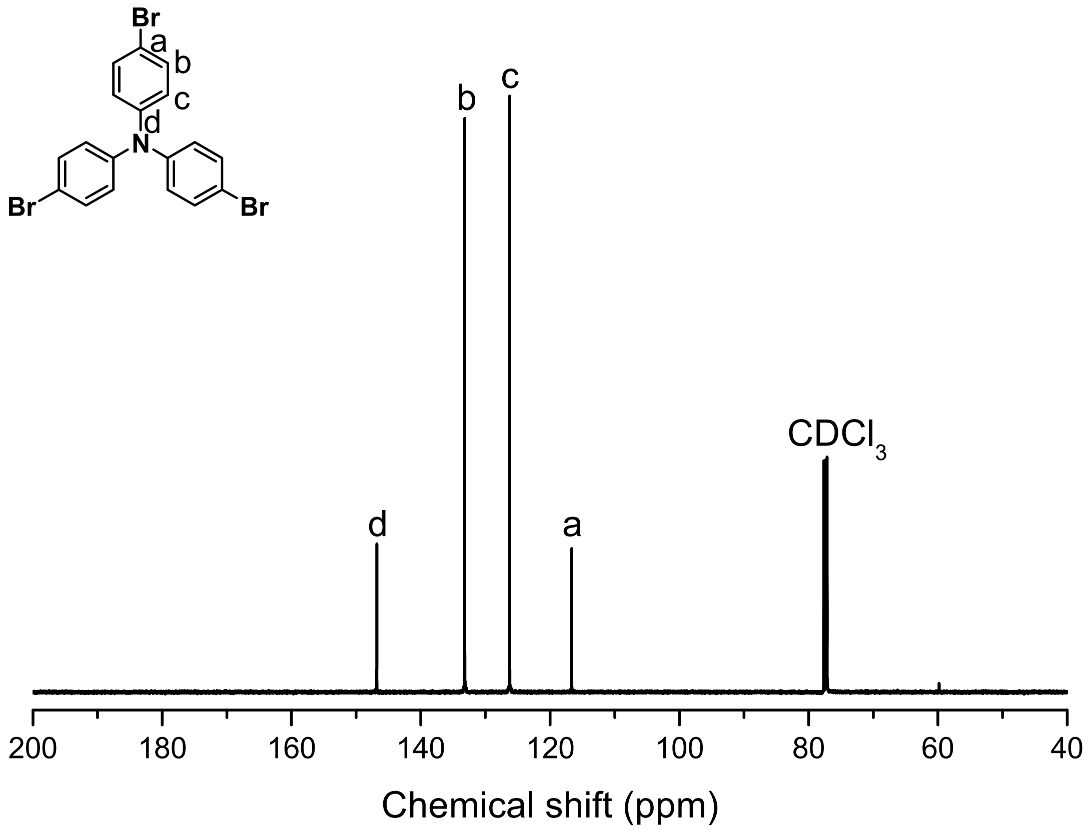

Figure S5. ^{13}C NMR spectrum of TPA-Br_3.

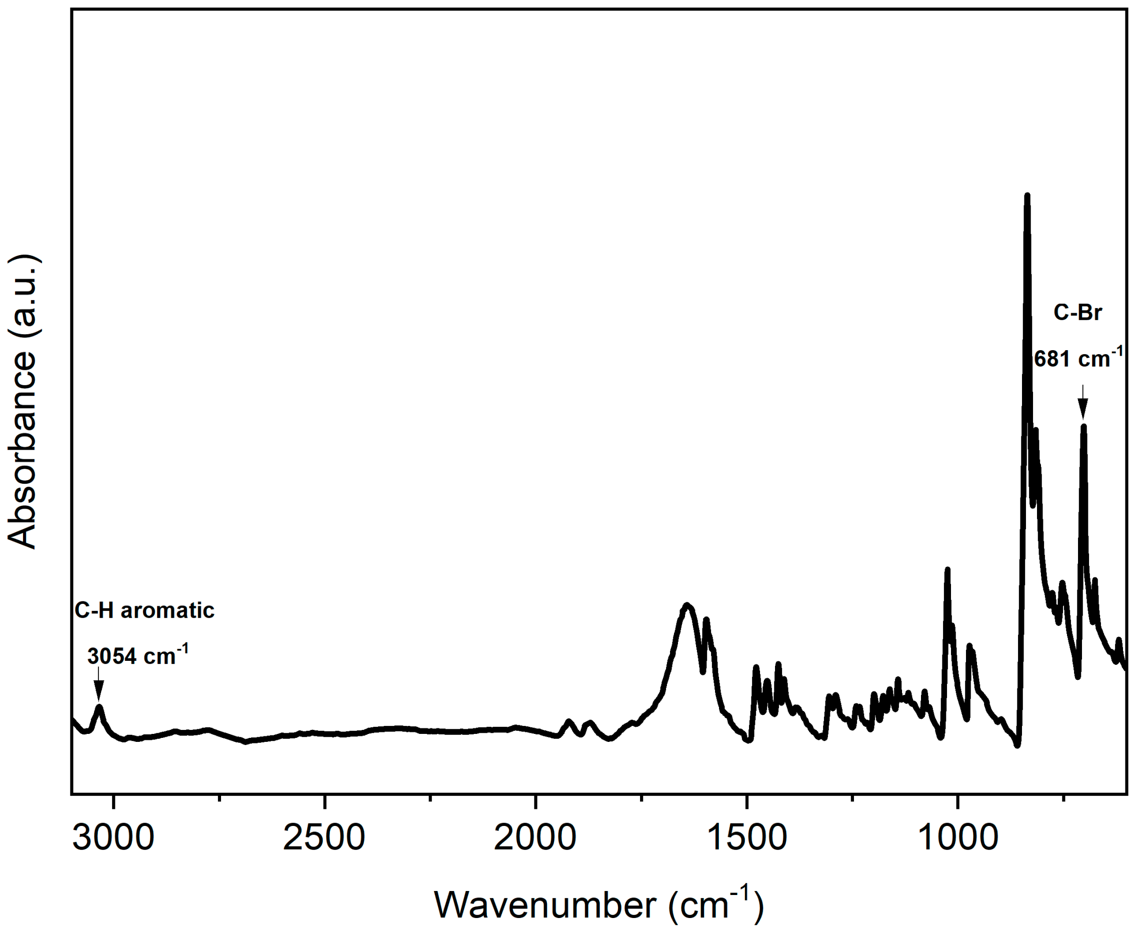

Figure S6. FTIR spectrum of Py-Br_4.

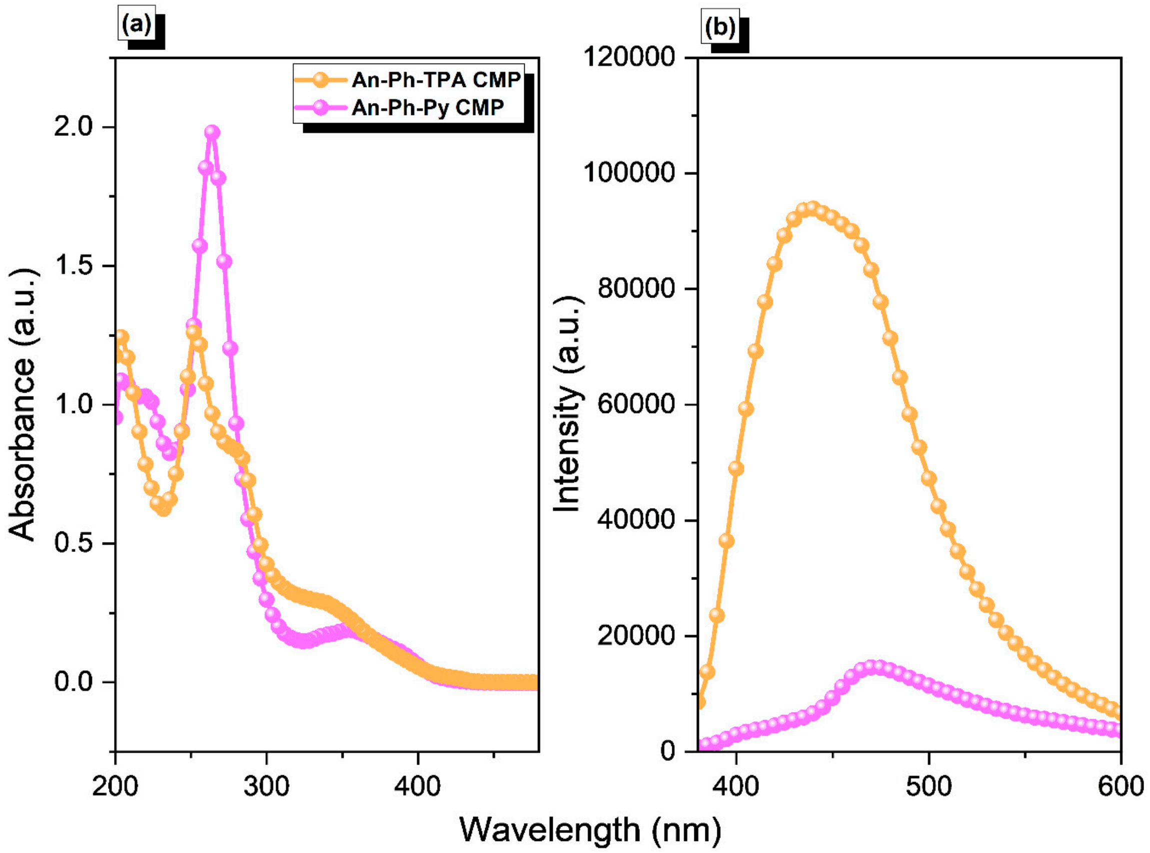

Figure S7. UV (a) and PL (b) profiles of An-Ph-TPA CMP and An-Ph-Py CMP.
